# Transcriptome Analysis, Machine Learning, and Experimental Identification of CDK7 Affecting the Progression of Pregnancy-induced Hypertension by Influencing Macrophage Polarization

**DOI:** 10.2174/0115665240384035250807100445

**Published:** 2025-08-22

**Authors:** Suyan Gu, Xiuqing Zhou, Xueyan Shen, Chunhui Xiao, Cuihong Gao, Xuan Zhang

**Affiliations:** 1 Department of Gynecology and Obstetrics, The Fourth Hospital of Shijiazhuang, No. 16, North Tangu Street, Chang 'an District, Shijiazhuang, 050000, People’s Republic of China

**Keywords:** Pregnancy-induced hypertension, immune, machine learning, CDK7, macrophage, gene set enrichment analysis

## Abstract

**Introduction:**

Pregnancy-induced hypertension (PIH) is a severe pregnancy complication characterized by placental insufficiency, abnormal vascular remodeling, and immune dysregulation, but personalized therapeutic markers remain unclear. This study aimed to identify key genes and explore immune mechanisms in PIH using transcriptome analysis, machine learning, and experimental validation.

**Methods:**

We analyzed the GSE204835 transcriptomic dataset to screen differentially expressed genes (DEGs) and performed Gene Ontology (GO), Kyoto Encyclopedia of Genes and Genomes (KEGG), Reactome, and Gene Set Enrichment Analysis (GSEA) for functional annotation. Immune infiltration analysis was also performed to examine the immune landscape in PIH. Least Absolute Shrinkage and Selection Operator (LASSO) regression identified key genes, which were validated in a PIH cell model. Flow cytometry and immunofluorescence assays assessed the effect of CDK7 knockdown on macrophage polarization.

**Results:**

A total of 1,598 DEGs (1,123 upregulated, 475 downregulated) were identified. Enrichment analyses highlighted associations with embryonic organ development, oxidative phosphorylation, angiogenesis, and oxidative stress. Immune infiltration analysis revealed altered eosinophil and macrophage polarization in PIH. LASSO regression selected 12 key genes, with CDK7 showing the most significant upregulation in the PIH model. CDK7 knockdown promoted macrophage polarization toward the anti-inflammatory M2 phenotype.

**Discussion:**

These findings link CDK7 to immune dysregulation in PIH by modulating macrophage polarization, expanding our understanding of PIH’s molecular mechanisms. The study’s limitations include reliance on public datasets and *in vitro* models, warranting *in vivo* validation.

**Conclusion:**

CDK7 emerges as a potential therapeutic target for PIH, offering new insights into immunoregulatory interventions for this complication.

## 1 INTRODUCTION

Pregnancy-induced hypertension (PIH) is a common and serious complication, affecting approximately 5% to 10% of pregnancies worldwide [1-3]. Its onset is influenced by multiple factors, including maternal age, obesity, ethnicity, a history of hypertension, and multiple pregnancies [4, 5]. PIH typically arises after 20 weeks of gestation, characterized by elevated blood pressure and, in some cases, increased urinary protein levels [6]. This condition presents significant risks to both maternal and fetal health, including placental dysfunction, preterm birth, fetal growth restriction, and severe maternal complications such as preeclampsia and eclampsia [7, 8]. Consequently, early detection and management are of paramount importance for minimizing its impact on maternal-fetal [9].

PIH’s pathological features primarily encompass placental insufficiency, abnormal vascular remodeling, and immune system dysregulation [10, 11]. These factors contribute to endothelial dysfunction and an increase in vasoconstrictive agents, which result in hypertension and compromise maternal-fetal health [12, 13]. Molecularly, PIH is closely associated with placental factors (*e.g.,* soluble fms-like tyrosine kinase-1 [sFlt-1] and soluble endoglin [sEng]), impaired angiogenesis, altered immune responses, and abnormal expression of specific genes [14, 15]. For instance, sFlt-1 inhibits angiogenesis by binding to vascular endothelial growth factor (VEGF), thereby inducing placental ischemia and exacerbating PIH clinical manifestations [16]. Moreover, emerging evidence indicates that immune dysregulation, manifested as macrophage polarization and immune cell activation, plays a crucial role in disease progression [17, 18]. Despite advancements, effective biomarkers for early diagnosis and therapeutic approaches remain limited, underscoring the necessity of precision medicine to improve patient outcomes.

The integration of bioinformatics and machine learning presents promising prospects for identifying PIH biomarkers and formulating early diagnostic strategies [19-21]. Bioinformatics tools can identify key genes and signaling pathways associated with the disease [22], elucidating the roles of placental factors and immune dysfunction [23]. Machine learning algorithms, capable of processing vast amounts of clinical and molecular data, can pinpoint genes or biomarkers closely linked to PIH pathogenesis, providing essential tools for diagnosis and management. Recent progress demonstrates that machine learning not only enhances the accuracy of early diagnosis but also facilitates the development of personalized treatment strategies tailored to individual genetic profiles [24]. Therefore, the combination of bioinformatics and machine learning holds substantial potential for advancing the understanding and clinical management of PIH.

In this study, we retrieved and analyzed a dataset on hypertensive disorders of pregnancy from the Gene Expression Omnibus (GEO) database. Employing a suite of bioinformatics tools, we explored the underlying biological functions associated with PIH development, its immune characteristics, and identified key genes involved in PIH through machine learning techniques. Additionally, we successfully established a cellular model to validate the expression of these genes and highlighted the role of cyclin-dependent kinase 7 (CDK7) in macrophage polarization and PIH progression.

## 2 MATERIALS AND METHODS

### 2.1 Transcriptome Data Download and Analysis

We retrieved the GSE204835 microarray data from the GEO database to identify differentially expressed genes (DEGs) in placental tissues from hypertensive pregnancies and control groups. Expression profiling data were mapped to gene names using the SangerBox ID conversion tool (http://sangerbox.com/). Differential expression analysis was performed with the R package limma, and Perl scripts were utilized to automate the process. DEGs were selected based on the criteria of *P* < 0.05 and log |FC| > 1. Data visualization was carried out using the ggplot2 and pheatmap packages.

### 2.2 GO, KEGG, and Reactome Functional Enrichment

To explore the biological functions and pathways associated with PIH, we used the BioWinford Platform (http://biowinford.site/) for annotation and visualization. DEGs were subjected to Gene Ontology (GO) and Kyoto Encyclopedia of Genes and Genomes (KEGG) pathway enrichment analyses. Additionally, Reactome pathway analysis was performed to further investigate the molecular pathways and reactions implicated in PIH. Genes with at least three overlapping entries and a *P* value ≤ 0.01 were considered statistically significant in all analyses.

### 2.3 GSEA Functional Enrichment Analysis

We conducted Gene Set Enrichment Analysis (GSEA) to investigate the differential expression of genes associated with PIH, focusing on GO, KEGG, and Reactome pathways. Gene lists, ranked by logFC or t-value, were input into the GSEA software. The analysis was performed with a genome size range of 15-500 and 1,000 permutations to ensure robustness. A significance threshold of *P* < 0.05 was applied to identify enriched gene sets. This approach facilitates the identification of biologically relevant pathways significantly associated with PIH, providing deeper insights into the underlying molecular mechanisms.

### 2.4 Immune Infiltration Analysis

To assess immune cell infiltration in PIH and normal controls, we utilized several algorithms from the BioWinford Platform (http://biowinford.site/). Immune cell type proportions were analyzed using the CIBERSORT, ESTIMATE, and xCell algorithms. These analyses were performed on normalized gene expression data to determine both relative and absolute immune cell abundances. The results were visualized using box plots to facilitate comparison of immune infiltration levels between the groups. Statistical significance was assessed using the Wilcoxon rank-sum test, with *P* < 0.05 considered significant.

### 2.5 Key Gene Selection in PIH Using Machine Learning (LASSO)

To identify key genes associated with PIH, we employed the Least Absolute Shrinkage and Selection Operator (LASSO) regression model, a widely used machine learning technique for variable selection. Gene expression data were normalized and used to train the LASSO model, which selects the subset of genes most predictive of the condition. The optimal regularization parameter (λ) was determined through 10-fold cross-validation to minimize overfitting and enhance the model's predictive accuracy. Genes selected by the model were considered potential candidates for further investigation in the context of PIH. Statistical significance was evaluated using cross-validation error, with a significance threshold of *P* < 0.05.

### 2.6 Culture of Cell

In this study, we utilized two key cell lines: HTR8-SVneo and THP-1. HTR8-SVneo, an immortalized human trophoblast cell line obtained from the SUNNCELL, preserves essential characteristics such as invasion, migration, and secretion, making it a widely used model in pregnancy-related disease research [25-28]. In this study, we employed the HTR8-SVneo cell line to construct a PIH model for exploring disease-related genes and pathways. THP-1, a human acute monocytic leukemia cell line purchased from the Shanghai Cell Bank of the Chinese Academy of Sciences, can be induced to differentiate into macrophages, which then release various cytokines. This cell line is commonly used in research on immune and inflammatory diseases [29-32]. In this study, we used THP-1 cells to investigate the effect of CDK7 on macrophage polarization and to analyze the immune mechanisms underlying PIH.

HTR8-SVneo and THP-1 cells were cultured in RPMI-1640 medium (Gibco, Thermo Fisher Scientific, Waltham, MA, USA; catalog number 11875-093), supplemented with 10% fetal bovine serum (FBS; Gibco, catalog number 16000-044) and 1% penicillin-streptomycin (Gibco, catalog number 15140-122). The cells were maintained at 37°C in a humidified atmosphere containing 5% CO₂. The medium was replaced every 2-3 days, and cells were passaged with 0.25% trypsin-EDTA (Gibco, catalog number 25200-072) when they reached approximately 80% confluence. All reagents used were sterile and of high quality to ensure optimal growth conditions.

### 2.7 Establishment of a Cell Model for PIH

To establish a cellular model mimicking PIH, we selected the HTR8-SVneo human trophoblast cell line due to its relevance to placental function. The cells were treated with angiotensin II (Ang II; Sigma-Aldrich, St. Louis, MO, USA; catalog number A9525) at a concentration of 10 nM for 24 hours to simulate vascular dysfunction. Additionally, cells were exposed to hypoxic conditions (1% O₂) for 24 hours to mimic placental ischemia, a condition resembling PIH.

### 2.8 Western blotting

Soluble fms-like tyrosine kinase 1 (sFlt-1) is a major anti-angiogenic factor secreted by trophoblast cells in the placenta. Its levels are significantly elevated in PIH, where it binds to vascular endothelial growth factor (VEGF), thereby inhibiting angiogenesis and contributing to disease symptoms [33-37]. Soluble endoglin (sEng), a coreceptor for transforming growth factor-beta (TGF-β) with anti-angiogenic properties, is produced by endothelial cells and trophoblasts. Its levels also rise in PIH, disrupting the TGF-β signaling pathway and promoting disease progression [38]. Both sFlt-1 and sEng act synergistically and are considered key biomarkers for PIH research and clinical diagnosis [39-41].

Western blotting was performed to detect PIH-related biomarkers sFlt-1 and sEng, validating the PIH model. Total protein was extracted from HTR8-SVneo cells under PIH simulation conditions using RIPA lysis buffer with protease inhibitors. Protein concentration was measured with the BCA Protein Assay Kit. Equal amounts of protein (20 μg) were separated by 10% SDS-PAGE and transferred to a PVDF membrane. After blocking with 5% non-fat milk, the membrane was incubated overnight at 4°C with primary antibodies for sFlt-1 (ab124737) and sEng (ab111477), followed by HRP-conjugated secondary antibody incubation at room temperature. Protein signals were detected using ECL detection reagent and imaged with the ChemiDoc MP Imaging System. GAPDH was used as an internal control, and densitometric analysis was conducted with ImageJ software for data quantification.

### 2.9 Real-time RT-PCR

To evaluate the gene expression levels of SYT17, NCSTN, CDK7, CRK, NIPAL1, MID1IP1, AKR1B15, ARSA, DAG1, KRAS, GTF2F1, and PPP4C in the PIH model and control groups, RT-qPCR was performed. Total RNA was extracted from HTR8-SVneo cells treated under PIH-like conditions using TRIzol reagent (Thermo Fisher Scientific, catalog no. 15596026), and cDNA was synthesized using the PrimeScript™ RT Reagent Kit (Takara Bio, catalog no. RR047A). Gene-specific primers were designed using Primer-BLAST (NCBI), with the following sequences: SYT17 (Forward: 5'-ACAGCCATCCTCGCTCAAG-3', Reverse: 5'-GCAAGGCAAGGAGAGAGAGG-3'); NCSTN (Forward: 5'-CAGGAGCCATGAGGAGGAGA-3', Reverse: 5'-ACACAGGAGGATGAGGGAAG-3'); CDK7 (Forward: 5'-TGTCTGGAGCCTCCTGAAG-3', Reverse: 5'-CAGTGGATAGGCAGAGGGA-3'); CRK (Forward: 5'-TTGGAGGAGGAAGCGAGT-3', Reverse: 5'-TGAGAGGAGATGAGGTGAG-3'); NIPAL1 (Forward: 5'-AGCGAGGAGCTGTGGTAGG-3', Reverse: 5'-AGTGGAAGGGAGGAAGGAG-3'); MID1IP1 (Forward: 5'-CGGAAAGGAGCGGAGGT-3', Reverse: 5'-CAGGAGGAAGGAGAGAGGAG-3'); AKR1B15 (Forward: 5'-GAAGAGAGCAGGAGGTGGA-3', Reverse: 5'-GGTGGAAGGAGGAAGGAAG-3'); ARSA (Forward: 5'-ATGACAGGTGAGGTAGAGG-3', Reverse: 5'-AGGGAAGAGGGAGGAAGGAG-3'); DAG1 (Forward: 5'-GGAAGGAAGGAGGAAGGA-3', Reverse: 5'-GAGGAGAGGAAGGAGGGA-3'); KRAS (Forward: 5'-TGAGGAGAGGAAGGAGGAAG-3', Reverse: 5'-AGGAGAGGAAGGAGGAGGA-3'); GTF2F1 (Forward: 5'-AAGAGGAGGAAGAGGAAGG-3', Reverse: 5'-GGAAGGAAGGAGGAAGGAG-3'); PPP4C (Forward: 5'-GAGGAGGAGGAAGAGGAGG-3', Reverse: 5'-AGGAAGGAAGAGGAGGAGG-3'). RT-qPCR was conducted using SYBR Green Master Mix (Applied Biosystems, catalog no. 4309155), with GAPDH as the internal control. The PCR conditions included an initial denaturation at 95°C for 10 minutes, followed by 40 cycles of denaturation at 95°C for 15 seconds, annealing at 60°C for 30 seconds, and extension at 72°C for 30 seconds. Relative gene expression was calculated using the 2^(-ΔΔCt) method, and comparisons were made between the PIH model and control groups.

### 2.10 Flow Cytometry

To examine the effect of CDK7 gene inhibition on macrophage polarization in a PIH model, HTR8-SVneo cells were transfected with two distinct small interfering RNA (siRNA) sequences targeting CDK7 (Santa Cruz Biotechnology, catalog number sc-37393) using Lipofectamine™ RNAiMAX Reagent (Thermo Fisher Scientific, catalog number 13778075). The siRNA sequences used were as follows: si-CDK7 #1: 5'-GAGCUUGUUGCAGGAGGAU-3' and si-CDK7 #2: 5'-GAAGCUUUCAAGGAGGAAU-3'. Control cells were transfected with a non-targeting siRNA (Santa Cruz Biotechnology, catalog number sc-37007). Following 48 hours of transfection, cells were exposed to Angiotensin II (Ang II, Sigma-Aldrich, catalog number A9525) for 24 hours to induce PIH-like conditions. After treatment, cells were collected and stained with antibodies targeting macrophage markers CD86 (FITC, BioLegend, catalog number 305606) and CD206 (PE, BioLegend, catalog number 321106). The stained cells were analyzed by flow cytometry (BD FACSCanto™ II, BD Biosciences), and the expression levels of CD86 and CD206 were quantified based on the percentage of positive cells and mean fluorescence intensity (MFI). The results were compared between the PIH model, CDK7 knockdown, and control groups to assess the role of CDK7 in macrophage polarization under PIH-like conditions.

### 2.11 Immunofluorescence

To investigate the impact of CDK7 gene inhibition on the macrophage polarization markers iNOS and CD206 in a PIH model, HTR8-SVneo cells were transfected with siRNA targeting CDK7 (Santa Cruz Biotechnology, catalog number sc-37393) using Lipofectamine™ RNAiMAX Reagent (Thermo Fisher Scientific, catalog number 13778075). The cells were subsequently treated with Angiotensin II (Ang II, Sigma-Aldrich, catalog number A9525) for 24 hours to induce PIH-like conditions. Following treatment, cells were fixed with 4% paraformaldehyde (Sigma-Aldrich, catalog number 158127) for 15 minutes and permeabilized with 0.2% Triton X-100 (Sigma-Aldrich, catalog number 9002-93-1) for 10 minutes. After blocking with 5% BSA (Sigma-Aldrich, catalog number A9647), the cells were incubated overnight at 4°C with primary antibodies against iNOS (Abcam, catalog number ab15323) and CD206 (Abcam, catalog number ab64693) at the appropriate dilutions. After washing, the cells were incubated with Alexa Fluor 488-conjugated goat anti-rabbit secondary antibody (Thermo Fisher Scientific, catalog number A11008) for 1 hour at room temperature, followed by nuclear staining with DAPI (Thermo Fisher Scientific, catalog number 62248). Fluorescence images were captured using a fluorescence microscope (Olympus IX83), and the expression levels of iNOS and CD206 were quantified by measuring fluorescence intensity and the percentage of positively stained cells using ImageJ software.

### 2.12 Statistical analysis

Statistical analysis was conducted using R software (version 4.3.1). The Student’s t-test was applied to compare normally distributed data, while the Wilcoxon rank-sum test was used for non-normally distributed data. For non-parametric data, results are presented as the median with interquartile range (IQR). A p-value of less than 0.05 was considered statistically significant for all tests.

## 3 RESULTS

### 3.1 Identification of Differentially Expressed Genes in PIH

To identify DEGs between the PIH and control groups, we retrieved the GSE204835 transcriptome dataset from the GEO database. This dataset comprises 12 placental samples from healthy controls and 36 placental samples from patients with PIH. Transcriptomic data were processed and analyzed using R and Perl software, along with the limma package. DEGs were determined using the thresholds of *P* < 0.05 and |logFC| > 1. To enhance the visualization of the results, we utilized the ggplot2 and pheatmap packages. The heatmap analysis identified a total of 1,598 DEGs in the PIH group compared to the controls, with 1,123 genes upregulated and 475 genes downregulated (Fig. **1**).

### 3.2 Functional Enrichment of Differentially Expressed Genes in PIH

To investigate the biological functions and pathways associated with DEGs in PIH, we performed functional enrichment analyses using the GO, KEGG, and Reactome databases.

GO enrichment analysis revealed significant associations between DEGs and biological processes such as embryonic organ development, urogenital system development, organ morphogenesis, mesenchymal development, sensory organ morphogenesis, meiotic cell cycle regulation, cytoplasmic translation, and the ubiquitin-dependent ER-associated degradation (ERAD) pathway (Fig. **2A**). KEGG pathway enrichment identified significant associations with cancer-related pathways (Fig. **2B**). Reactome analysis further highlighted DEGs involved in the regulation of slit-robo signaling, eukaryotic translation initiation, cap-dependent translation initiation, L13a-mediated translational silencing, GTP hydrolysis, ribosomal subunit ligation, SRP-dependent co-translational protein targeting, free 40S subunit pool formation, EIF2AK4 (GCN2) response to amino acid deficiency, nonsense-mediated decay (independent of exon junction complex), and viral mRNA translation (Fig. **2C**).

Additionally, GSEA was conducted to provide further insight into functional pathways. GSEA revealed significant enrichment of DEGs in processes such as angiogenesis, vascular development, negative regulation of transcription by RNA polymerase, response to hypoxia, and response to reactive oxygen species (Fig. **3A**). KEGG pathway enrichment *via* GSEA identified significant associations with Alzheimer's disease, oxidative phosphorylation, Parkinson’s disease, protein processing in the endoplasmic reticulum, and ribosome function (Fig. **3B**). Reactome functional enrichment analysis highlighted key pathways such as antigen processing and cross-presentation, cytokine signaling in the immune system, developmental biology, the ER-phagosome pathway, and hemostasis (Fig. **3C**).

Finally, enrichment analysis using the Molecular Signatures Database (MsigDB) revealed significant enrichment of DEGs in various biological processes, including adipogenesis (*P* = 2e-04) (Fig. **4A**), apical junctions (*P* = 1e-04) (Fig. **4B**), apoptosis (*P* = 0) (Fig. **4C**), coagulation (*P* = 0.0143) (Fig. **4D**), complement activation (*P* = 0.0173) (Fig. **4E**), DNA repair (*P* = 1e-04) (Fig. **4F**), epithelial-mesenchymal transition (*P* = 1e-04) (Fig. **4G**), late estrogen response (*P* = 0.0041) (Fig. **4H**), fatty acid metabolism (*P* = 0.0052) (Fig. **4I**), G2/M checkpoint regulation (*P* = 0.0084) (Fig. **4J**), tissue hypoxia (*P* = 1e-04) (Fig. **4K**), IL-2/STAT5 signaling (*P* = 0.0081) (Fig. **4L**), IL-6/JAK/STAT3 signaling (*P* = 0.004) (Fig. **4M**), KRAS signaling (*P* = 0.0293) (Fig. **4N**), and mitotic spindle assembly (*P* = 0.0016) (Fig. **4O**).

### 3.3 Analysis of Immune Correlates in Hypertension During Pregnancy

Recent studies have highlighted the intricate role of immune cells in the progression of various diseases, including PIH [5, 42-44]. To explore the influence of DEGs in PIH on immune cell dynamics and identify potential therapeutic targets, we applied three distinct immunoassay algorithms.

First, CIBERSORT analysis revealed significant differences in immune cell composition between the hypertensive pregnancy group and the normal control group, particularly in eosinophil and macrophage polarization (Fig. **5A**). ESTIMATE analysis further confirmed notable differences in immune cell infiltration, stromal scores, and tumor purity between the PIH and control groups (Fig. **5B**). Additionally, using the xCELL algorithm, we identified significant variations in the expression of several immune cell types, including conventional dendritic cells (cDCs), dendritic cells (DCs), endothelial cells, hepatocytes, and hepatic stellate cells (HSCs) between the two groups. Other cell types, such as immature dendritic (iDC) cells, memory B-cells, and neuronal cells, also exhibited differential expression between the hypertensive and control groups (Fig. **5C**).

### 3.4 Machine Learning Identification of Key Genes Associated with the Development of PIH

To identify key genes involved in the progression of hypertensive disorders during pregnancy, we conducted λ-based LASSO regression analysis. This approach enabled us to select 12 genes most strongly associated with the development of PIH (Fig. **6A**). We then visualized these 12 genes, which included SYT17, NCSTN, CDK7, CRK, NIPAL1, MID1IP1, AKR1B15, ARSA, DAG1, KRAS, GTF2F1, and PPP4C (Fig. **6B**). These genes may serve as potential molecular markers, providing insights into the pathogenesis of PIH and offering opportunities for the identification of novel therapeutic targets and biomarkers for disease monitoring.

### 3.5 Construction of the PIH Model and Validation of Key Genes

To further explore the potential role of key genes in the progression of hypertensive disorders during pregnancy, we constructed a cellular model of PIH. HTR8-SVneo cells were treated with angiotensin II and hypoxia, and the expression of PIH-related markers, sFlt-1 and sEng, was assessed to validate the model. Our results showed a significant increase in both sFlt-1 and sEng protein levels in the model group, confirming that the model accurately replicated the pathological features of PIH (Fig. **7A**-**7B**).

After validating the model, we examined the expression of key genes identified through bioinformatics and machine learning approaches, including SYT17, NCSTN, CDK7, CRK, NIPAL1, MID1IP1, AKR1B15, ARSA, DAG1, KRAS, GTF2F1, and PPP4C, using RT-qPCR. Our analysis revealed that the expression of SYT17, CDK7, CRK, NIPAL1, AKR1B15, ARSA, DAG1, KRAS, GTF2F1, and PPP4C was significantly upregulated in the model group, with CDK7 showing the most pronounced increase (Fig. **8A**-**8J**). In contrast, the expression of NCSTN and MID1IP1 exhibited only minor, statistically insignificant changes (Fig. **8K**, **8L**). These findings further support the involvement of these key genes in the pathogenesis of PIH, with CDK7 emerging as a particularly significant marker.

### 3.6 CDK7 Knockdown in the PIH Cell Model Promotes Macrophage Polarization Toward the M2 Phenotype

In our previous bioinformatics analysis, we observed significant changes in macrophage polarization in PIH, and emerging evidence suggests that CDK7 is involved in macrophage polarization in inflammatory arthritis [45, 46]. To further investigate the involvement of CDK7 in macrophage polarization in the context of PIH, we conducted flow cytometry analysis on a co-culture system consisting of the PIH cell model, the CDK7-knockdown PIH cell model, and macrophages. Our findings revealed that CDK7 knockdown resulted in a significant reduction in the expression of the M1 macrophage marker CD86, while the M2 macrophage marker CD206 was significantly upregulated in the CDK7-knockdown PIH cell model (Fig. **9A**-**9B**).

Furthermore, immunofluorescence staining confirmed these results, showing a marked decrease in the M1-type macrophage marker iNOS and a significant increase in the M2-type macrophage marker CD206 in the CDK7-knockdown PIH model (Fig. **9C**). These findings suggest that silencing CDK7 in the PIH cell model promotes a shift in macrophage polarization toward the anti-inflammatory M2 phenotype, highlighting the potential immunomodulatory role of CDK7 in the pathophysiology of PIH.

## 4 DISCUSSION

Pregnancy-induced hypertension (PIH) is a common and serious pregnancy complication, affecting approximately 5% to 10% of pregnant women worldwide [47-49]. Despite significant advancements in understanding the mechanisms underlying PIH, challenges persist in the early diagnosis of the disease, the identification of reliable therapeutic biomarkers, and the development of personalized treatment strategies [50, 51]. In this study, we employed a range of bioinformatics tools and machine learning techniques to comprehensively investigate the biological functions, immune characteristics, and key genes involved in PIH. We began by downloading transcriptomic datasets related to PIH from the GEO database and performing differential expression analysis to identify genes and signaling pathways most closely associated with PIH pathogenesis. Additionally, machine learning algorithms were used to identify key genes strongly correlated with PIH progression, providing potential candidates for therapeutic biomarkers. We also established a cellular model of PIH to validate the expression of these key genes. Notably, we focused on the role of the CDK7 gene in PIH progression and found that CDK7 likely contributes to PIH development by modulating macrophage polarization, suggesting its potential as a therapeutic target in PIH management.

In our study, we identified 1,598 DEGs in PIH using the GSE204835 dataset. Our findings are consistent with previously described molecular mechanisms of PIH and contribute to a deeper understanding of its pathophysiology. For example, earlier transcriptomic analyses of PIH placental samples have highlighted altered gene expression in key processes such as angiogenesis, immune response, and vascular remodeling—results that align closely with those of our study. Of the DEGs identified, 1,123 were upregulated and 475 were downregulated, supporting the hypothesis that PIH is strongly associated with placental dysfunction, vascular abnormalities, and immune system alterations. Notably, the upregulation of anti-angiogenic factors such as sFlt-1 and sEng plays a critical role in exacerbating PIH by impairing placental development.

Our functional enrichment analysis further revealed that the DEGs are significantly associated with biological processes such as organ morphogenesis, mesenchymal development, and angiogenesis, which are characteristic of PIH as a disorder of placental development and vascular remodeling. These findings reinforce the established view that PIH is associated with abnormal vascular development and placental insufficiency [52]. Additionally, our analysis identified significant enrichment in pathways related to cell cycle regulation, cytoplasmic translation, and protein degradation, including the ubiquitin-dependent endoplasmic reticulum-associated degradation (ERAD) pathway. These results offer new insights into the molecular mechanisms driving PIH progression. Previous studies have also linked the dysregulation of protein degradation pathways, particularly in placental trophoblast cells, to PIH pathogenesis, underscoring the critical role of these processes in disease development [53].

Interestingly, our analysis also revealed significant enrichment of DEGs in cancer-related pathways, such as translation initiation and ribosome function regulation. This suggests that the cellular stress responses and translational dysregulation observed in cancer may also contribute to the placental pathology associated with PIH. These findings are consistent with prior research highlighting the role of cellular stress and protein translation dysfunction in PIH development. Together, our results offer new potential targets for therapeutic intervention, particularly within translational regulation and protein degradation pathways, which could inform future PIH treatments.

Another notable finding from our analysis is the significant enrichment of DEGs in immune-related pathways, particularly those involved in cytokine signaling, antigen processing, and immune cell cross-presentation. Recent studies have emphasized the crucial role of the immune system in PIH, with particular focus on the imbalance between M1- and M2-type macrophage polarization [54]. Our observation of enriched immune-related pathways further reinforces the critical role of immune dysregulation in the pathogenesis of PIH, suggesting that immune modulation could serve as a promising therapeutic strategy for managing PIH.

Additionally, the enrichment of pathways related to angiogenesis and vascular development reinforces the crucial role of abnormal angiogenesis in PIH. Well-established placental factors, such as sFlt-1 and sEng, are known to exacerbate placental ischemia and elevate blood pressure by inhibiting VEGF-mediated angiogenesis. Our findings, which reveal significant enrichment of DEGs in pathways associated with endothelial cell function and angiogenesis, provide additional support for the role of these molecular mechanisms in the pathophysiology of PIH. These results not only enhance our understanding of the disease but also validate these molecular pathways as potential biomarkers for early diagnosis and therapeutic targeting in PIH.

The impact of transcriptional dysregulation in the progression of various diseases cannot be overstated. For example, studies have shown that mutations leading to the inactivation of the p53 gene and the overexpression of MDM2 significantly disrupt normal transcriptional networks, thereby driving tumorigenesis [55]. Cyclin-dependent kinase 7 (CDK7), a core component of transcriptional regulation, forms the CDK-activating kinase (CAK) complex with cyclin H and MAT1, which regulates the phosphorylation of RNA polymerase II, thereby mediating the key step of protein-coding gene transcription [56, 57]. Although significant progress has been made in understanding the role of CDK7 in basal transcription and in various diseases [58-61], its role in PIH remains unclear. Previous studies have observed abnormal transcriptional pathways and downregulation of CAK complex genes in the placenta of preeclamptic patients [62], but the specific molecular mechanisms have not been deeply explored. In contrast, our study is the first to demonstrate a significant upregulation of CDK7 in a PIH cellular model, suggesting a more complex and previously unexplored role for CDK7 in the pathogenesis of PIH. 

Research on rheumatoid arthritis has shown that CDK7 inhibitors can reduce RNA polymerase II pausing, suppress pro-inflammatory gene expression, and reverse macrophage polarization [45]. However, the regulatory mechanisms of CDK7 in PIH are markedly different. Our study is the first to confirm that CDK7 not only participates in transcriptional regulation in PIH but also mediates immune imbalance by regulating macrophage polarization. Experiments conducted in a PIH cellular model demonstrate that knockdown of CDK7 facilitates the polarization of macrophages toward the anti-inflammatory M2 phenotype. The imbalance between M1 and M2 macrophages is a key factor in triggering placental inflammation and vascular dysfunction in PIH and is also a major pathogenic contributor to various inflammatory diseases [63]. This finding reveals the central role of CDK7 in reshaping the inflammatory microenvironment of PIH and suggests that CDK7 could serve as a potential target for macrophage-mediated immune modulation in PIH therapy.

Earlier studies have established the critical role of immune cell dysregulation in various inflammatory diseases, including PIH. For instance, in glaucoma research, it was found that arterial stiffness is associated with abnormal vascular and immune function in glaucoma patients, suggesting that improving vascular and immune function could aid in glaucoma treatment [64]. However, our study is the first to identify CDK7 as a potential immune-regulatory molecule involved in PIH, broadening the scope of CDK7 research in inflammatory diseases and offering new avenues for targeted therapy in PIH.

It is noteworthy that our study identified several differentially expressed genes, including SYT17, NCSTN, CDK7, CRK, NIPAL1, MID1IP1, AKR1B15, ARSA, DAG1, KRAS, GTF2F1, and PPP4C. These genes may play a coordinated role in the pathogenesis and progression of PIH by regulating biological processes such as angiogenesis, cell migration, structural maintenance, metabolism, stress response, and transcriptional regulation. Although this study primarily focuses on the function of CDK7, the molecular mechanisms involving the other genes should not be overlooked.

A core pathological feature of PIH is abnormal placental angiogenesis and remodeling. NCSTN is a critical component of the γ-secretase complex involved in the processing of Notch receptors [65]. The Notch signaling pathway is essential for regulating cell fate and tissue development [66, 67]. Studies have shown that during placental development, Notch signaling plays a pivotal role in the proliferation, differentiation, and invasiveness of trophoblast cells, and its dysregulation can lead to abnormal placental vascular development [68-71]. Abnormal expression of NCSTN may disrupt Notch signaling, impairing normal placental vascular development. KRAS, a key member of the Ras/MAPK signaling pathway, plays a critical role in regulating cell proliferation, differentiation, and survival. Studies indicate that activation of KRAS is associated not only with rapid cell proliferation but also with the formation of abnormal vascular networks. Furthermore, research suggests a potential link between KRAS and CDK7 in transcriptional regulation. CDK7 influences gene transcription by regulating the phosphorylation of RNA polymerase II, and the expression of KRAS may be influenced by transcriptional regulation [72, 73], which could jointly impact the pathological progression of PIH. Although research on SYT17 has primarily focused on the nervous system, its involvement in cell membrane fusion and signaling pathways may also regulate vascular-related functions in the placental microenvironment, though its exact role remains to be further elucidated.

Beyond angiogenesis, cell migration and structural maintenance are also essential for normal placental development. CRK, an adaptor protein, is involved in cell adhesion, movement, and signal transduction [74]. Elevated expression of CRK may enhance the migratory ability of trophoblast cells, whereas suppression of its expression may limit their invasion into the decidua, thus hindering the remodeling of spiral arteries. MID1IP1, through its interaction with the microtubule-associated protein MID1, regulates microtubule stability [75, 76]. Dysfunction in MID1IP1 may impact the polarity and structural integrity of placental cells. MID1IP1 cooperates with MID1 to stabilize microtubules and is involved in cell processes affected by microtubule stability. Dysfunction of MID1IP1 can lead to changes in trophoblast cell morphology and polarity, affecting placental architecture. Additionally, MID1IP1 is involved in fatty acid metabolism [77], suggesting it may serve as a bridge connecting metabolic states and cellular structure, thus influencing placental morphogenesis. DAG1, a key extracellular glycoprotein, is crucial for cell migration and epithelial polarization [78]. Abnormal expression of this gene may weaken the adhesion and signaling between trophoblast cells and the extracellular matrix, disrupting cell polarity and ordered migration. This structural and functional disturbance may affect the placental invasion of maternal vessels, leading to impaired placental vascular remodeling and PIH.

In terms of metabolism and stress regulation, AKR1B15, a member of the aldoketoreductase family, primarily regulates intracellular redox balance [79]. Upregulation of its expression may reflect an oxidative stress environment in PIH placental tissue, a known key mechanism in the pathogenesis of PIH [80]. ARSA, encoding arylsulfatase A, is involved in sphingolipid metabolism and lysosomal function [81]. Elevated ARSA levels may indicate a repair process in placental tissue following cellular damage. NIPAL1, a trans-membrane protein regulating intracellular magnesium ion homeostasis, affects gene expression related to cell adhesion, damage, angiogenesis, inflammation, coagulation, and growth [82]. Given magnesium’s impact on vasodilation and vascular smooth muscle function, abnormal expression of NIPAL1 may indirectly affect placental hemodynamics and contribute to a hypertensive state.

Regarding transcriptional regulation and cell cycle control, GTF2F1, a key subunit of the RNA polymerase II initiation complex, regulates gene transcription initiation and stability [83]. Its expression changes in the placenta could alter the transcription of critical genes, affecting cell differentiation and functional maintenance. PPP4C, the catalytic subunit of protein phosphatase 4, dephosphorylates various substrate proteins, regulating their activity. This dephos-phorylation process plays a key role in cell signaling, cell cycle control, and stress responses [84], which may impact placental tissue renewal and vascular stability.

In summary, while this study primarily focuses on CDK7, the differentially expressed genes identified may each contribute individually to the pathological progres-sion of PIH through mechanisms such as angiogenesis regulation, cell migration, structural maintenance, metabolic stress response, and transcriptional/cell cycle control. These genes likely interact within a complex network to collectively regulate placental homeostasis and contribute to pathological changes. Future research should investigate the regulatory interactions among these key genes to uncover a more comprehensive molecular understanding of PIH and provide a theoretical foundation for targeted therapeutic strategies.

Additionally, our study highlights the significant upregulation of CDK7 in PIH and its potential dual role in both transcriptional regulation and immune modulation, particularly in macrophage polarization. These findings offer new insights and therapeutic possibilities for managing PIH.

## CONCLUSION

This study investigates the molecular mechanisms underlying pregnancy-induced hypertension (PIH), identifying key genes and immune-related pathways associated with the disease. Utilizing bioinformatics tools and machine learning techniques, the research reveals a significant upregulation of CDK7 in the placenta of women with PIH. The study highlights CDK7’s dual role in transcriptional regulation and immune modulation, particularly in macrophage polarization. These findings suggest that CDK7 may serve as a potential therapeutic target for PIH, offering new insights for the development of more effective immunomodulatory treatments.

## LIMITATIONS

Several limitations should be acknowledged in this study. First, the data primarily originated from publicly available transcriptomic datasets in the GEO database. However, the samples may not fully capture the genetic and phenotypic diversity of PIH patients across different ethnicities, regions, and clinical subtypes, which limits the generalizability of our findings. Second, our cellular model of PIH, used for gene expression validation and CDK7 exploration, lacks the complex *in vivo* physiological environment and cell-cell interactions. *In vivo* validation through animal models or clinical trials is necessary to confirm the roles of the identified genes and pathways in PIH. The absence of such evidence restricts the translation of our results to clinical practice. Finally, while bioinformatics and machine learning have identified several potentially important genes and pathways, our understanding of their specific mechanisms in PIH remains preliminary. Further in-depth mechanistic studies are required to elucidate their interactions and contributions to PIH pathogenesis.

## Figures and Tables

**Fig. (1):**
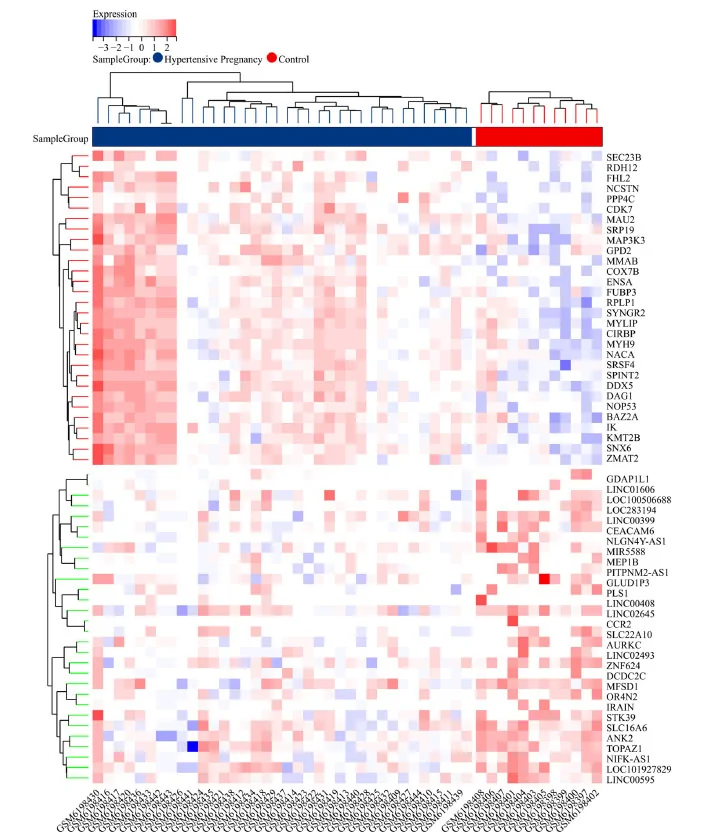
Identification of differential genes in PIH. A heatmap illustrating the differential expression of genes between the PIH group (blue) and controls (red).

**Fig. (2):**
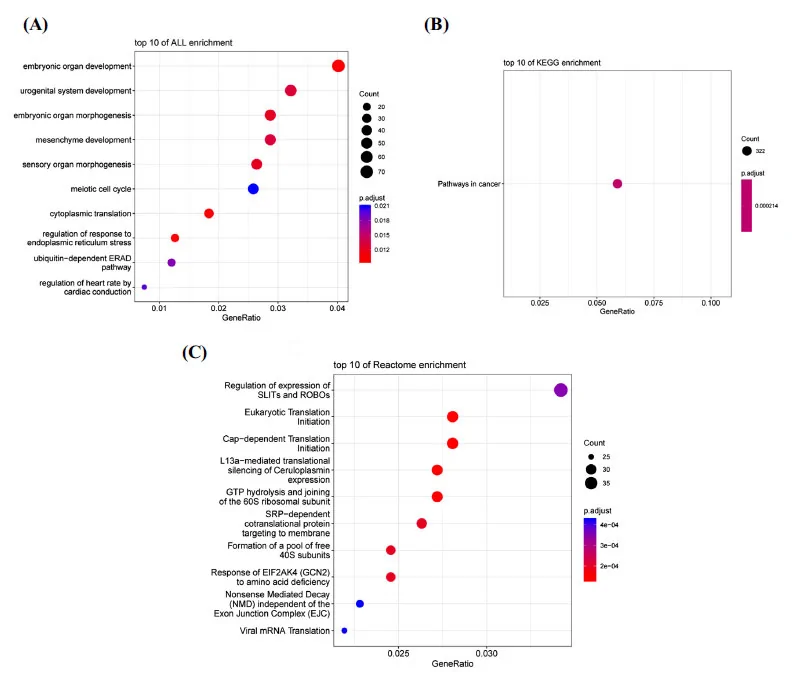
Functional enrichment of hypertensive differential genes in PIH. (**A**) Bubble plot depicting the GO functional enrichment analysis of differential genes in PIH. (**B**) Bubble plot illustrating functional enrichment analysis of KEGG in PIH. (**C**) Bubble plot presenting the Reactome pathway enrichment analysis of differential genes in PIH.

**Fig. (3):**
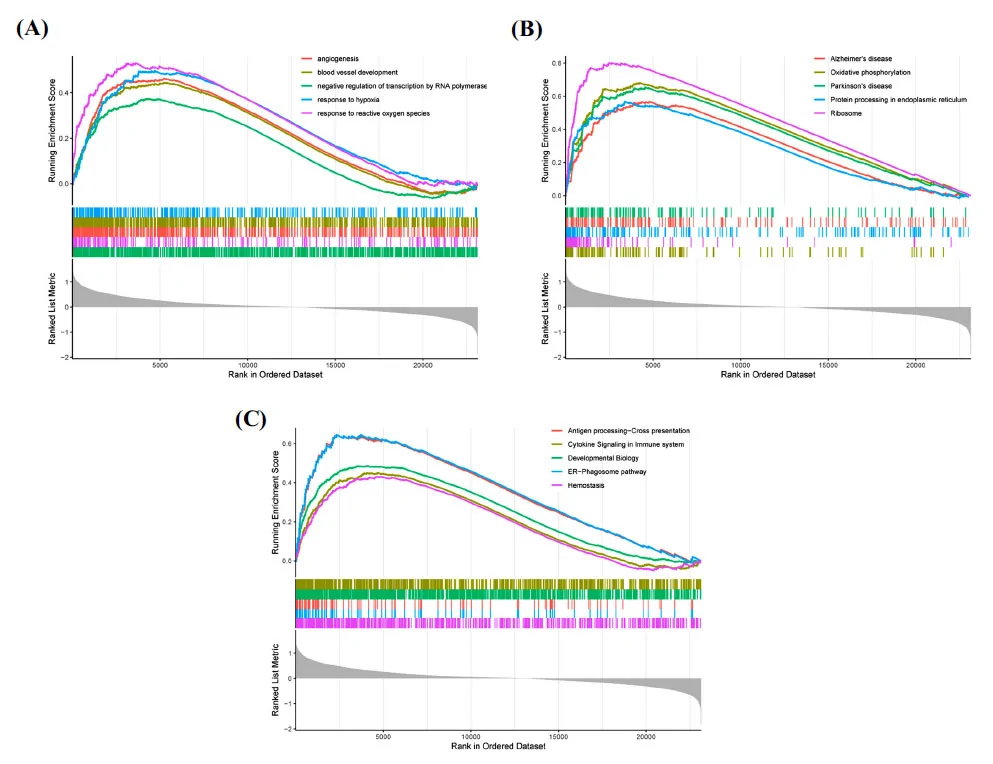
Functional enrichment of GSEA analysis of PIH differential genes. (**A**) Bubble plot showing functional enrichment analysis of GO in PIH. (**B**) Bubble diagram showing functional enrichment analysis of KEGG in PIH. (**C**) Bubble diagram showing functional enrichment analysis of Reactome in PIH.

**Fig. (4):**
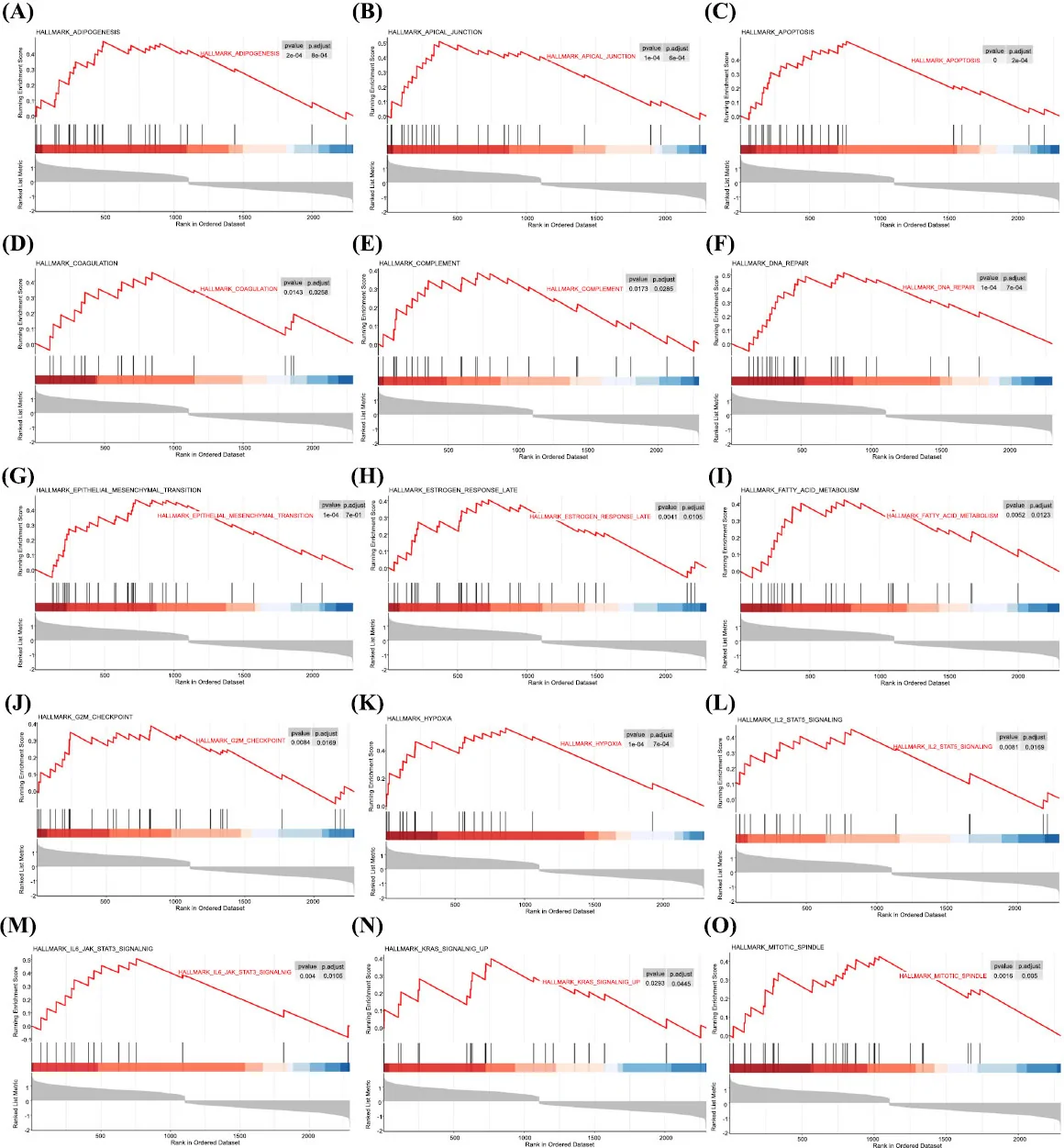
Enrichment analysis of the Molecular Signatures Database (MsigDB) for hypertensive differential genes in pregnancy. (**A**) Adipogenesis. (**B**) Apical junctions. (**C**) Apoptosis. (**D**) Coagulation. (**E**) Complement activation. (F) DNA repair. (**G**) Epithelial-mesenchymal transition. (**H**) Late estrogen response. (**I**) Fatty acid metabolism. (**J**) G2/M checkpoint regulation. (**K**) Tissue hypoxia. (**L**) IL-2/STAT5 signaling. (**M**) IL-6/JAK/STAT3 signaling. (**N**) KRAS signaling. (**O**) Mitotic spindle assembly.

**Fig. (5):**
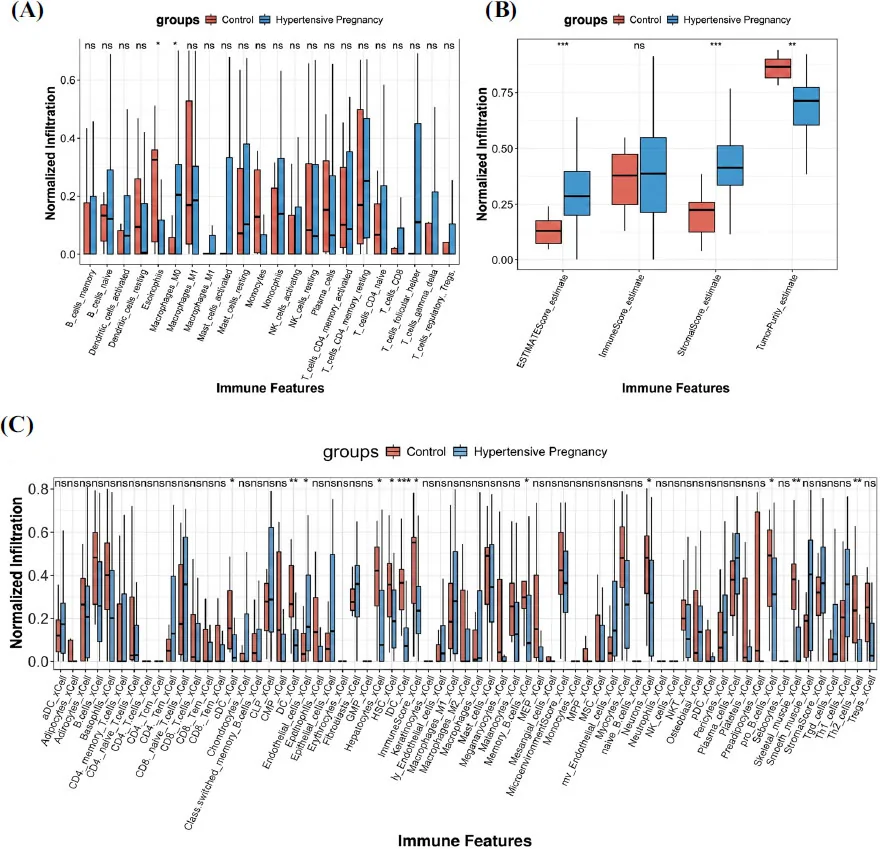
Immune cell correlation analysis of the hypertensive group and normal control group during pregnancy. (**A**) Cibersort algorithm. (**B**) ESTIMATE algorithm. (**C**) xCELL algorithm. (Blue: PIH group, red: control group) * *p* < 0.05, ** *p* < 0.01, *** *p* < 0.001, and **** *p* < 0.0001.

**Fig. (6):**
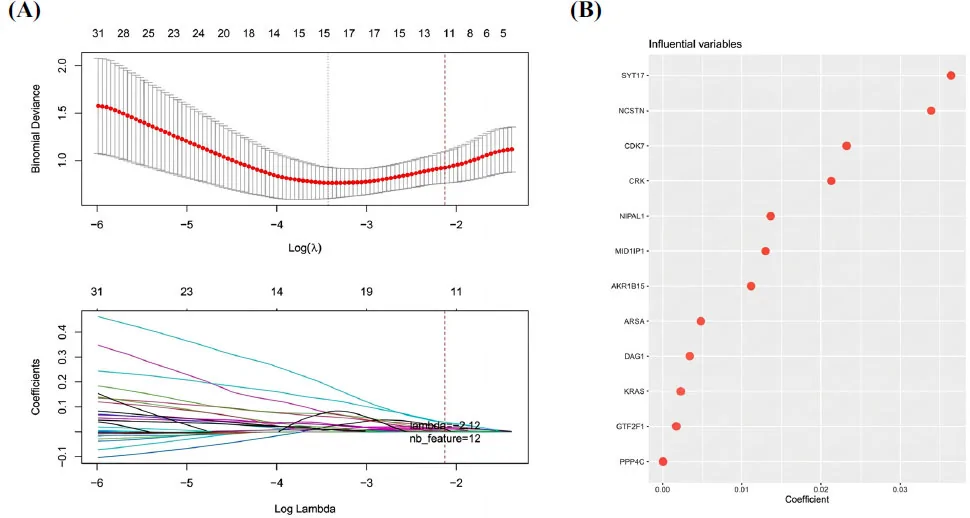
PIH key genes identified using machine learning. (**A**) Parameter selection by LASSO regression. (**B**) Illustration of LASSO coefficient curves for selected factors.

**Fig. (7):**
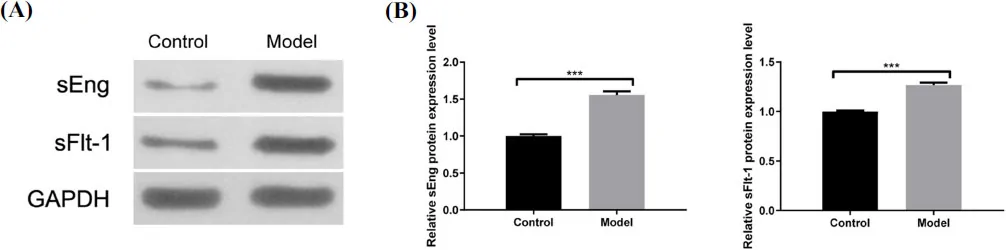
Validation of a cellular model of PIH. (**A**) Western blotting for sFlt-1 and sEng protein levels. (**B**) Quantitative analysis of sFlt-1 and sEng protein expression. *** *p* < 0.001.

**Fig. (8):**
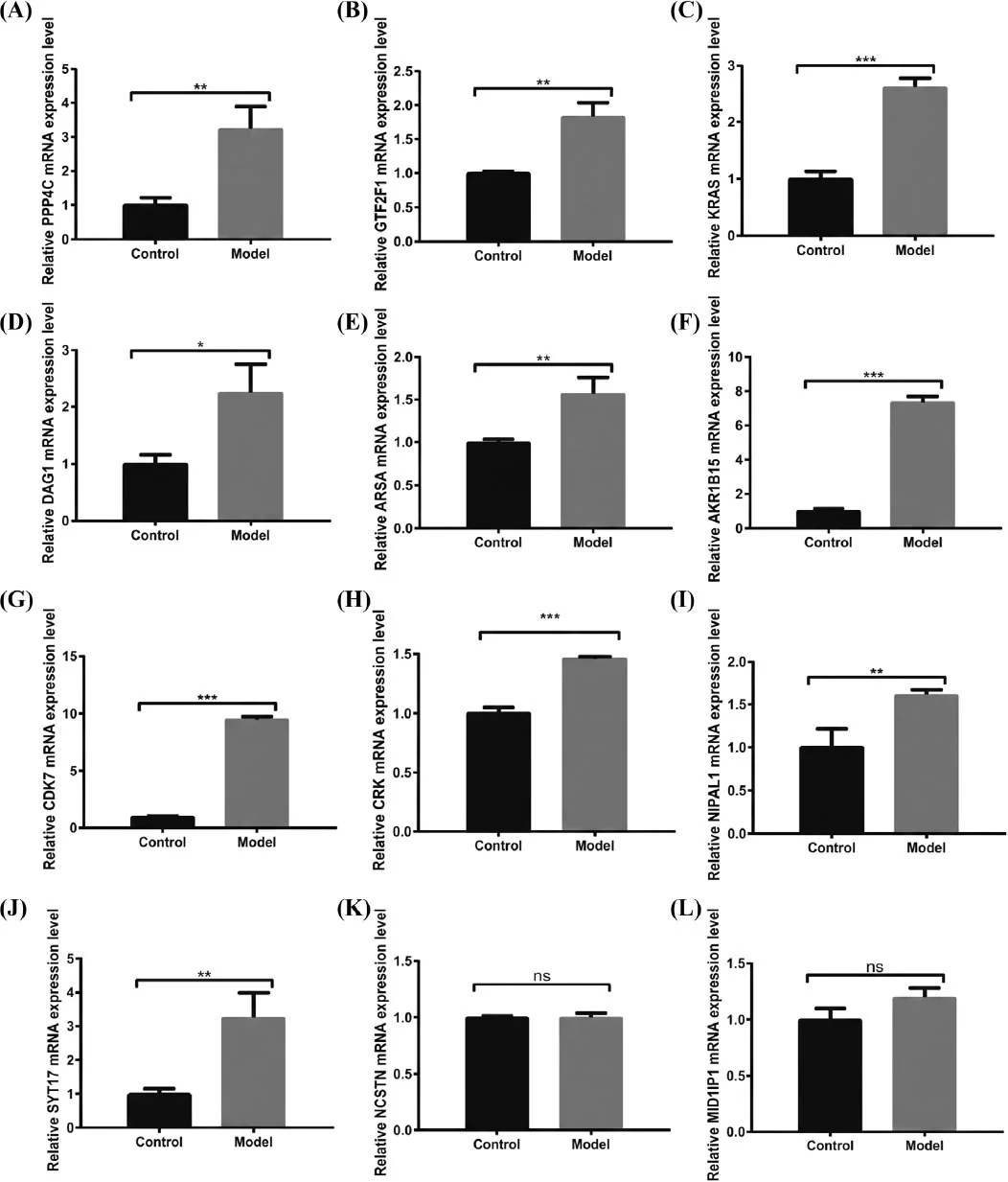
RT-qPCR validation of key genes in a cellular model of PIH. (A) PPP4C.(**B**) GTF2F1. (C) KRAS. (**D**) DAG1. (**E**) ARSA. (**F**) AKR1B15. (**G**) CDK7. (**H**) MID1IP1. (**I**) NIPAL1. (**J**) CRK. (**K**) NCSTN. (L) SYT17. ** *p* < 0.01 and *** *p* < 0.001.

**Fig. (9):**
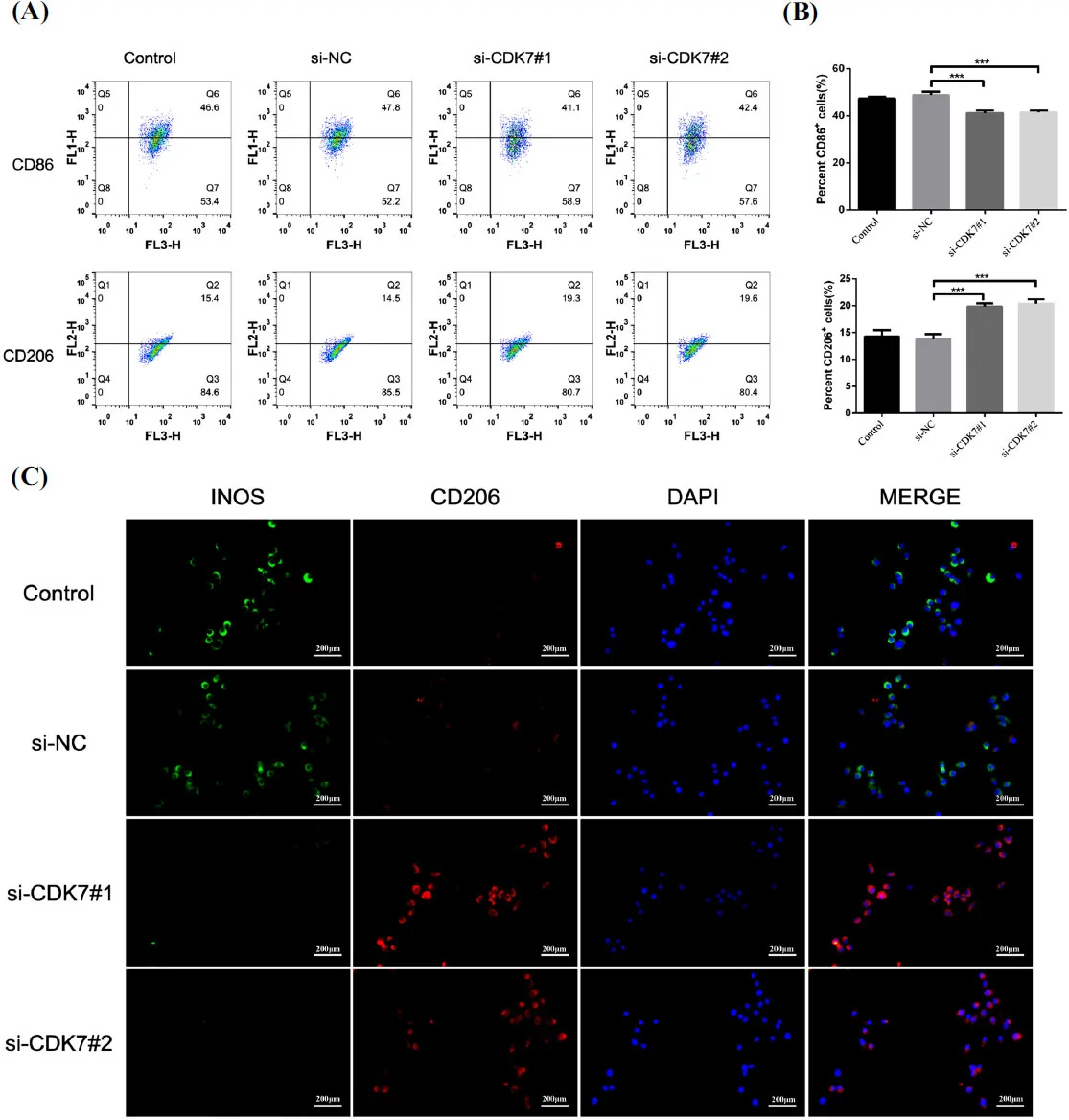
Promotion of macrophage polarization to M2-type after si-CDK7. (**A**) Flow cytometry detection of CD86, CD206 expression after si-CDK7. (**B**) Statistical analysis by flow cytometry. (**C**) Immunofluorescence detection of INOS and CD205 expression after si-CDK7. *** *p* < 0.001.

## Data Availability

The GEO public dataset used in this study can be found in the data repository under the following accession number: GSE204835 Transcriptome Data.

## LIST OF ABBREVIATIONS

AKR1B15: Aldo-keto reductase 1B15
Ang II: Angiotensin II
ARSA: Arylsulfatase A
CAK: CDK-activating kinase
CDK7: Cyclin-dependent kinase 7
CRK: Adaptor protein CRK
DAG1: Dystroglycan 1
DEGs: Differentially expressed genes
ESTIMATE: Estimation of STromal and Immune cells in MAlignant Tumors using Expression data
FBS: Fetal bovine serum
FITC: Fluorescein isothiocyanate
GEO: Gene Expression Omnibus
GSEA: Gene Set Enrichment Analysis
GO: Gene Ontology
GTF2F1: General transcription factor II F subunit 1
iNOS: Inducible nitric oxide synthase
KEGG: Kyoto Encyclopedia of Genes and Genomes
KRAS: Kirsten rat sarcoma viral oncogene homolog
LASSO: Least Absolute Shrinkage and Selection Operator
MId1IP1: Midline 1 interacting protein 1
MsigDB: Molecular Signatures Database
NIPAL1: NIPA-like 1
NCSTN: Nicastrin
PE: Phycoerythrin
PIH: Pregnancy-induced hypertension
PPP4C: Protein phosphatase 4 catalytic subunit
qRT-PCR: Quantitative real-time polymerase chain reaction
sEng: Soluble endoglin
sFlt-1: Soluble fms-like tyrosine kinase-1
siRNA: Small interfering RNA
SYT17: Synaptotagmin 17
VEGF: Vascular endothelial growth factor
